# Taxonomic Revision of *D. carlosvilelai* Vela and Rafael, 2001, and Updated Record of *D. paraguayensis* Duda, 1927 Registry in Ecuador †

**DOI:** 10.3390/insects16090944

**Published:** 2025-09-09

**Authors:** Coraima Elizabeth Suárez, Ana Peñafiel-Vinueza, Violeta Rafael, María Isabel Tamayo, Doris Vela

**Affiliations:** Facultad de Ciencias Exactas, Naturales y Ambientales, Pontificia Universidad Católica del Ecuador, Av. 12 de octubre 10-76 y Ramón Roca, Quito 170525, Ecuador; coraima1993@hotmail.es (C.E.S.); adpvpiccola@gmail.com (A.P.-V.);

**Keywords:** *Drosophila*, Pasochoa Protected Forest, new species description, taxonomic revision, *tripunctata* group

## Abstract

*Drosophila carlosvilelai* and *D. paraguayensis* are species that were collected in the Pasochoa Protected Forest between 1996 and 1997. *D. carlosvilelai* was described incompletely as a new species from Ecuador. Regarding *D. paraguayensis*, recent collections have raised doubts about the validity of its records in Ecuador. Specifically, a new species found in the province of Napo fully matches the specimens of *D. paraguayensis* recorded in the Pasochoa Protective Forest. However, this new species does not match the description of *D. paraguayensis* by Vilela and Bächli. The collected specimens, preserved in alcohol, were analyzed at the Laboratorio de Genética Evolutiva. *D. carlosvilelai* was re-described based on the newly collected specimens. The species previously misidentified as *D. paraguayensis* is described as *D. paraloewi* sp. nov. and compared with *D. paraguayensis*, *D. loewi*, and *D. hemiloewi*. This analysis revealed that the aedeagus of *D. paraloewi* shares the dorsal structure of triangular projection on the aedeagus with *D. loewi* and *D. hemiloewi*; this morphological similarity suggests a closer phylogenetic relationship between them, rather than *D*. *paraguayensis*.

## Simple Summary

The present article aims to revise the taxonomic status and expand the morphological description of two species of *Drosophila* in Ecuador. The first one is *D. carlosvilelai*, whose original description lacks vital information for its accurate identification; therefore, we provide an updated re-description of this species, adding the description of its female, based on specimens collected from a newly recorded locality. The second one is *D. paraguayensis*, which was registered in 2001 at Pasochoa Protected Forest. However, this registry is not conclusive, because the characteristics used to identify this species were not described in either its original publication or its later re-description. Further taxonomic analysis indicates that the specimen is not *D. paraguayensis* but represents a previously undescribed species, which is formally described in this study as a new species, appearing to be closely related to *D. loewi*.

## 1. Introduction

The *Drosophila tripunctata* group comprises 89 species globally, of which 37% have been recorded in Ecuador [1]. In the country, these species are predominantly found in Andean cloud forests, which are classified as global biodiversity hotspots [2]. These ecosystems are currently under threat due to anthropogenic activities; therefore, taxonomic research is crucial for providing the data necessary for the conservation of both species and their ecosystems [3]. Studies on *Drosophila* species in Ecuador began in 1987 [4]. Since then, several new species from different neotropical groups have been discovered, and to date, 46 species of the *tripunctata* group have been identified, 13 of them are still waiting to be added to an Indexed Species list [1]. The species *D. carlosvilelai* Vela and Rafael, 2001, and *D. paraguayensis* Duda, 1927, both of which are members of the *tripunctata* group, were described based on specimens collected in the Pasochoa Protected Forest between 1996 and 1997 [5]. However, the original description of *D. carlovilelai* lacks the data on metric characters [5,6]; consequently, this deficiency in taxonomic data hinders accurate species identification when new specimens are encountered outside the type locality, due to the absence of essential morphological and systematic information required for reliable classification.

In the case of *D. paraguayensis*, its record from Ecuador may have a taxonomic discrepancy, due to misidentification: the specimens from the Pasochoa Protected Forests and others from the Napo Province externally appear to be *D. paraguayensis* but distinctively differ in the detailed structures of male terminalia, according to the re-description made by Vilela and Bächli [7]. These newly collected specimens, since 2018, across various regions of the country, present in the surstylus 8 (8–9) prensisetae and 8–9 (9–16) outer setae, but no inner setae. While Vela and Rafael [5] showed a greater number of prensisetae and outer setae. However, this new specimen does not conform to the morphological characteristics of *D. paraguayensis* re-described by Vilela and Bächli [7] or to the original description by Duda [8].

This work therefore provides a re-described description for *D. carlosvilelai,* detailing its morphological characteristics, including metric characters and the first description of the female, essential for accurate species identification. Furthermore, one of the specimens previously collected from Ecuador and misidentified as *D. paraguayensis* has been reidentified and now described as a new species, *Drosophila paraloewi* sp. nov.

## 2. Materials and Methods

The flies were collected in the Carchi and Pichincha provinces of Ecuador; the collection points were within El Ángel Ecological Reserve and the Pasochoa Protected Forest, respectively. The collections from the Pasochoa Protected Forest (00°28′52.6″ S 78°29′49.4″ W) were conducted between 1996 and 1997, at an elevation ranging from 3260 to 3310 m above sea level (masl). This protected forest corresponds to an ecosystem of the humid montane forest [9]. However, the collections from El Ángel Ecological Reserve (00°22′52.6″ S 78°09′44.4″ W) were carried out between 2016 and 2018, at an altitude of 3400 and 4200 masl. The Ecological Reserve corresponds to a paramo of Frailejones (*Espeletia* spp., Asteraceae) characterized by rosette-shaped leaves covered with whitish hairs [10]. The collected specimens of *Drosophila* were analyzed and preserved in 75% alcohol solution in the Laboratorio de Genética Evolutiva at Quito, Ecuador. For the male specimens, the last two abdominal segments of each fly were detached from the body under a stereomicroscope (Olympus SZ61, Tokyo, Japan). These segments were immersed in a 10% KOH solution and placed in a water bath for 8 min to remove the soft tissues and facilitate the dissection of the genital structures: epandrium, hypandrium, and aedeagus [11]. These structures were soaked in 100% glycerol on a plate for microscopic observation. The remaining body parts of each specimen were preserved in a glycerol–alcohol solution (95% ethanol and 5% glycerol) for subsequent mounting.

The morphological features of the terminalia from each specimen were compared with those of previously identified specimens, as well as with the taxonomic literature sourced from peer-reviewed journals and indexed databases. The structures were then illustrated using a camera lucida (Zeiss -47 46 20 9900) attached to a microscope (Zeiss -46 70 86). Measurements of the genital structures to calculate the indices were performed using a Zeiss Discovery V8 microscope (Oberkochen, Germany) with a built-in camera and Axio Vision V4 software. The guidelines and terminology proposed by Bächli et al. [6] were followed for measuring dry-mounted bodies and describing species. The index values for the paratypes and individual specimens examined as additional material are provided in parentheses in the description section.

We designed a neotype for *D. carlosvilelae* due to the loss of the holotype described and the poor quality of conservation of the paratypes from the work of Vela and Rafael [8]. These accord with the ICZN code.

The neotype, allotype, holotype, paratypes, and additional specimens examined have been deposited in the Museum of Zoology, Invertebrates Section, at the Pontificia Universidad Católica del Ecuador (QCAZ-I).

## 3. Results

This work has been registered in Zoobank with the following code: 6BADD1F7-97BF-4ED0-A5DD-F3A70830ACB9. Additionally, new nomenclature acts, i.e., designation of a neotype for *D. carlosvilelai* and the description of *D. paraloewi* sp. nov., undertaken in this work, are registered in Zoobank. Their respective codes can be found in their descriptions of this publication, in compliance with the rectification of the nomenclature code, carried out in 2012 by the International Commission on Zoological Nomenclature.

### 3.1. Re-Description of Drosophila carlosvilelai Vela and Rafael, 2001

Zoobank code: DE7E4B19-99FF-4E54-99E5-9E8823ADC650

**Figure 1:**
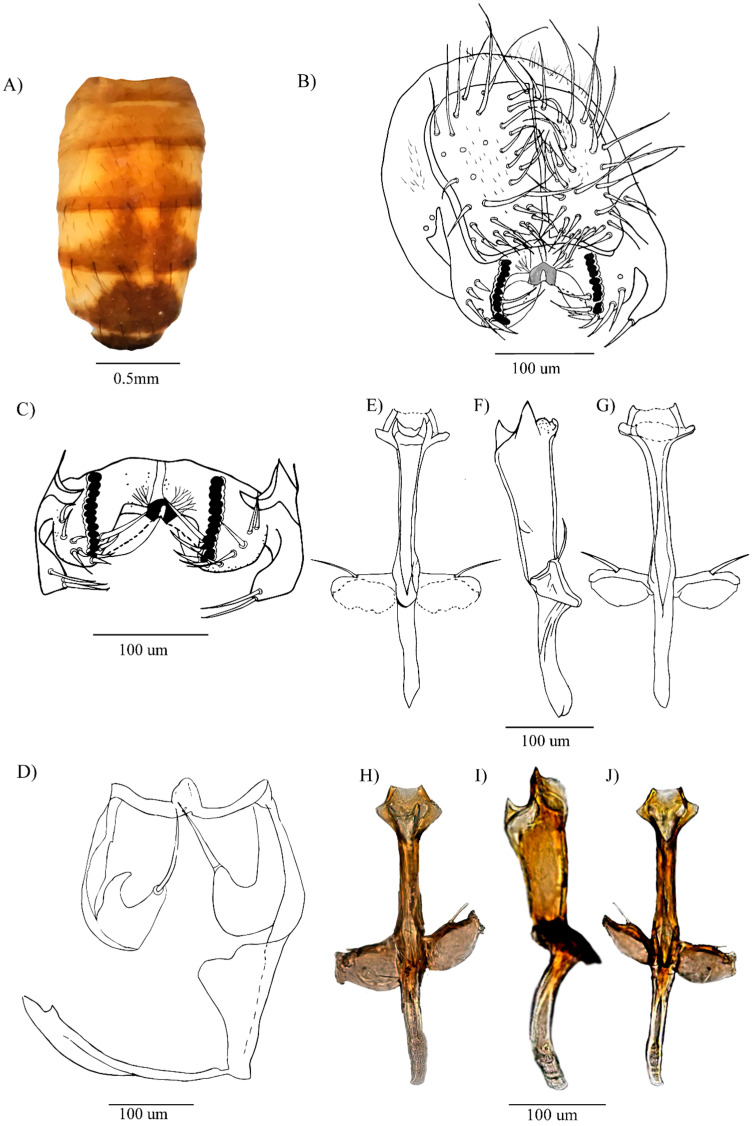
*Drosophila carlosvilelai,* neotype ♂ (QCAZ-I 261143): (**A**) Abdomen in dorsal view. (**B**) External terminalia. (**C**) Surstylus (drawn from an additional specimen collected from the type locality). (**D**) Hypandrium. (**E**–**G**) Aedeagus in dorsal, lateral, and ventral views, respectively. (**H**–**J**) Pictures of the aedeagus in dorsal, lateral, and ventral views, respectively.

**Figure 2:**
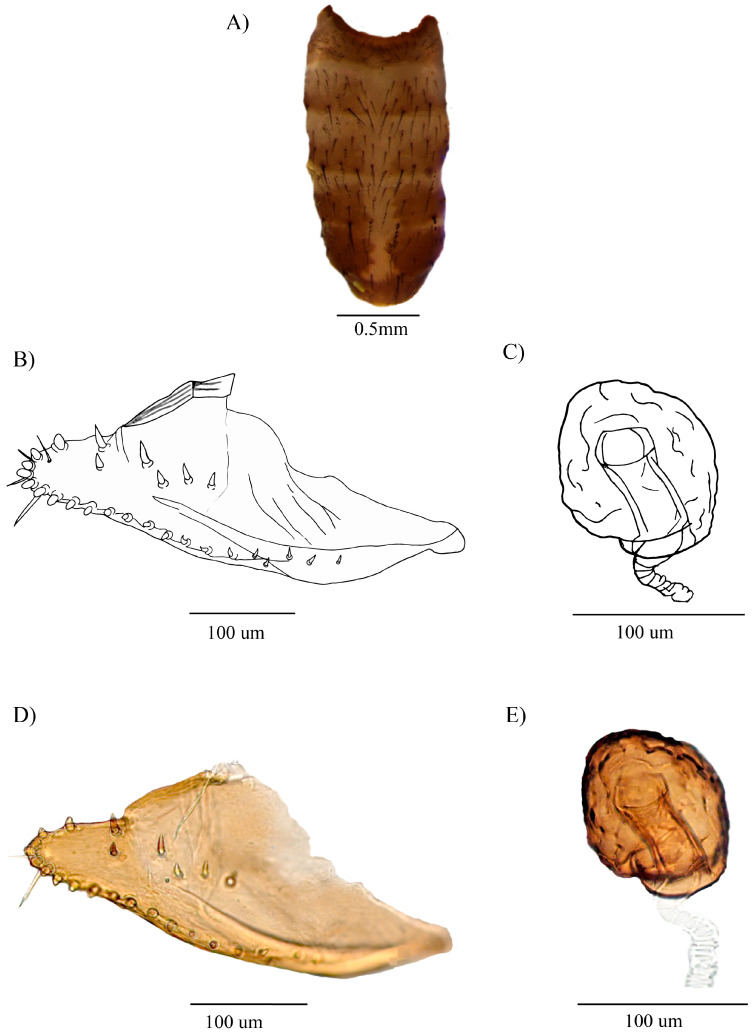
*Drosophila carlosvilelai*, allotype ♀ (QCAZ-I 260751): (**A**) Abdomen in dorsal view. (**B**) Oviscapt. (**C**) Spermatheca. (**D**) Picture of the oviscapt. (**E**) Picture of the spermatheca.

Figure 1A–J and Figure 2A–E

*Drosophila carlosvilelai* Vela and Rafael, (2001:100), incomplete description, no data on metric characters.

Type species. Neotype 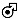 (dissected, terminalia in microtube, dry-mounted), Ecuador, Napo, Laguna de Papallacta, 3400 masl, 00°22′52.6″ S 78°09′44.4″ W, Nov. 2016, K. Casal et al., V. Rafael, A. Peñafiel, and C. Suárez det. (QCAZ-I 261143). Allotype 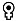 (dissected, terminalia in microtubes, dry-mounted), Ecuador, Carchi, Reserva Ecológica el Ángel, 3762 masl, 00°47′22.1″ N 77°54′03.2″ W, Jul. 2016, A. Peñafiel col., V. Rafael, A. Peñafiel, and C. Suárez det. (QCAZ-I 260751).

Additional specimens examined, 28 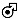 (dissected, terminalia in microtubes, dry-mounted), Ecuador, Napo, Laguna de Papallacta, 3400 masl, same data as the neotype, Nov. 2016, K. Casal col., V. Rafael, A. Peñafiel, and C. Suárez det. (QCAZ-I 261144-261170). 3 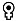 (dissected, terminalia in microtubes, dry-mounted, from an isofemale line), Ecuador, Carchi, Reserva Ecológica el Ángel, 3762 masl, same data as the allotype, A. Peñafiel col., V. Rafael, A. Peñafiel, and C. Suárez det. (QCAZ-I 260752-260754).

#### 3.1.1. Diagnosis

Head with yellow ocelli and slightly brown outlines. Wine-red eyes. Wing with a faintly shaded dM-Cu. Yellow abdomen with a dorsal midline from the second to the fourth tergite. Epandrium with a striated surstylus; chitinized decasternum with a more chitinized triangular central structure and two striated lateral projections. Aedeagus membranous ventro-medially, and refractive points, with three chitinized, apically pointed projections laterally and dorso-medially. Female: Abdomen yellow, with a medial dorsal stripe from the second to fifth tergite. Elongated, slipper-shaped oviscapt. Chitinized spermathecae, globose, striated on surface.

#### 3.1.2. Re-Description of Male

External appearance in the neotype: total length (body + wings) 4.67 (3.78–5.13) mm, body length 2.78 mm. Body color yellowish brown.

**Head.** Yellow brown. Frontal length 0.35 (0.25–0.36) mm, frontal index 0.88 (0.63–1.03), frontal tapering ratio 1.38 (1.08–1.50). Orbital plate yellow, median orbital seta shorter and slightly close to the outer edge of the orbital plate, vt index 2.70 (0.36–2.75); ratio or1/or3 1.06 (0.27–1.46), ratio or2/or1 0.17 (0.15–0.46). Ocellar triangle yellow, ocelli yellow with slightly brown outlines; frontal vitta, gena, and postgena yellow, cheek index 6.86 (4.18–11.00). Carina yellow not prominent, not furrowed. Palps yellow. One oral seta prominent, vibrissal index 0.69 (0.78–1.21). Eyes wine-red, eye index 1.20 (1.04–1.69). Arista with five dorsal and two ventral branches, forked terminal, and fine hairs.

**Thorax.** Yellowish brown, with six rows of acrostichal setulae between dorsocentral setae; h index 3.20 (0.69–3.80); dc index 0.64 (0.49–0.79). Scutellum slightly paler than the remainder of the thorax; apical scutellar setae convergent; scut index 1.39 (0.54–1.31). Median katepisternal seta slightly smaller than the anterior one, sterno index 1.05 (0.48–3.38).

**Wing.** Length 3.34 mm and a width of 1.44 mm; DM-Cu slightly shaded. Wing indices: wing = 2.32, C = 3.27, ac = 2.50, hb = 0.14, 4c = 0.62, 4v = 1.19, 5x = 1.25, M = 0.35, and prox. x = 0.28.

**Abdomen.** Yellow with dorsal midline from the second to the fourth tergite. Tergite 1 with a slightly brown band along the lower edge. Tergites 2–4 each two triangular brown spots that fade laterally. Tergites 5 and 6 each with a brown spot (Figure 1A).

**External terminalia.** Epandrium microtrichose; ventral lobe with 2 (1–3) setae. Free cerci. Surstylus with 12 (11–12) prensisetae and 9 (6–9) outer setae on both sides. Surstylus striated (Figure 1C). Chitinized decasternum with more chitinized triangular central processes and two striated lateral projections (Figure 1B,C).

**Internal terminalia**. Shield-shaped hypandrium with slightly chitinized dorsal arch, and yellow gonopods each bearing long seta (Figure 1D). Aedeagus chitinized, tubular, widened apically, membranous ventro-medially, with refractive points, with two chitinized projections laterally and dorso-medially (Figure 1E–J).

#### 3.1.3. Description of the Female

External appearance same as the male, except for the abdomen (Figure 2A).

**Abdomen.** Yellow with a dorsal midline from the second to the fifth tergite. Tergite 1 with a slightly brown band along the lower edge; tergites 2–5 with two triangular brown spots that fade laterally; tergite 6 with a brown spot (Figure 2A).

**Terminalia.** Oviscapt elongated, slipper-shaped with 6 inner ovisensilla with a rounded tip, one long, slightly curved trichome, and three small trichomes within inner ovisensilla, 6 discal and 15 marginal outer ovisensilla (Figure 2B,D). Spermathecae chitinized, globose, striated on surface (Figure 2C,E).

#### 3.1.4. Distribution

Known from the type locality, in the Pasochoa Protected Forest in Pichincha province, ranging from 3260 to 3310 masl. Laguna de Papallacta, in Napo province, at 3400 masl. El Ángel Ecological Reserve in Carchi province, at 3762 masl in Ecuador.

#### 3.1.5. Biology

Unknown. The examined specimens were collected using traps baited with fermented bananas. This species has been successfully cultured on a banana yeast medium [12].

#### 3.1.6. Note

The re-description was based on the specimens obtained from a new locality, Laguna de Papallacta. According to the ICZN, a neotype was designed for a complete description of this species, because the holotype described by Vela and Rafael [5] was not located at QCAZ-I, and the paratypes were in poor condition to designate a neotype from them.

### 3.2. Description of Drosophila paraloewi sp. nov.

Zoobank code: 6EB51C98-083E-4C2E-955A-5F8E6F07E363

Figure 3A–I

*Drosophila paraguayensis:* sensu Vela & Rafael 2001, (66), misidentification, not *Drosophila paraguayensis* Duda, 1927.

Holotype 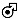 (dissected, terminalia in microtube, dry-mounted), Ecuador, Pichincha, Pasochoa, 3260–3310 masl, 00°28′ S 78°29′ W, July 1997, D. Vela col., V. Rafael, D. Vela, and C. Suárez det. (QCAZ-I 261171).

Paratypes 13 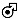 (dissected, terminalia in microtubes, dry-mounted), Ecuador, Pichincha, Pasochoa, 3260–3310 masl, same data as the holotype, D. Vela col., V. Rafael, D. Vela, and C. Suárez det. (QCAZ-I 261172-261183).

#### 3.2.1. Diagnosis

Arista with five dorsal and four ventral branches, forked terminal, and fine hairs. Median katepisternal seta slightly larger than the anterior katepisternal seta. Wings faintly yellow. Epandrium microtrichose with the ventral lobe bearing 1–2 setae near the insertion of the surstylus, without lower setae. Hypandrium with microtrichose gonopod bearing a long seta; paraphysis attached to the gonopod, with a small seta. Aedeagus distally widened, with a pair of chitinized, serrated laminae, dorso-apically bifurcated membrane bearing refractive points resembling a serrated edge, dorsally with a chitinized triangular, bifurcate rod-like structure (tpb), and with a globose striate membrane ventrally.

#### 3.2.2. Description of the Male

External appearance of the holotype: total length (body + wings) 5.96 (3.96–5.93) mm, body length 2.85 mm. Body color dark brown.

**Head**. Brown. Frontal length 0.36 (0.28–0.37) mm, frontal index 0.88 (0.78–1.33), frontal tapering ratio 1.44 (1.31–2.00); Orbital plate brown, anterior reclinate orbital seta close to proclinate orbital seta, vt index 2.57 (0.92–2.57); or1/or3 ratio 0.62 (0.66–1.07), or2/or1 ratio 0.75 (0.17–0.50). Ocellar triangle dark brown, ocelli dark yellow; frontal vitta, gena, and postgena yellowish brown, cheek index 6.89 (5.55–14.80). Vibrissa prominent, vibrissal index 0.33 (0.33–0.61). Carina dark brown, slightly prominent, not furrowed. Palpus brown. Wine-red eyes, eye index 1.13 (1.13–1.72). Arista with five dorsal and four ventral branches, a forked terminal branch, bifurcated, and with fine hairs.

**Thorax.** Brown, with six rows of acrostical setulae between anterior dorsocentral setae; h-index 0.76 (0.69–2.42); dc-index 0.67 (0.33–0.65). Apical scutellar setae convergent, scut index 1.16 (0.71–2.26). Median katepisternal seta slightly larger than the anterior katepisternal, sterno index 1.33 (0.70–1.33).

**Wings.** Slightly yellow; length 3.62 mm, width 1.44 mm. Wing indices: wing = 2.51, C = 4.86, ac = 1.84, hb = 0.35, 4c = 0.38, 4v = 0.81, 5x = 1.06, M = 0.22, and prox. x = 0.26.

**Figure 3:**
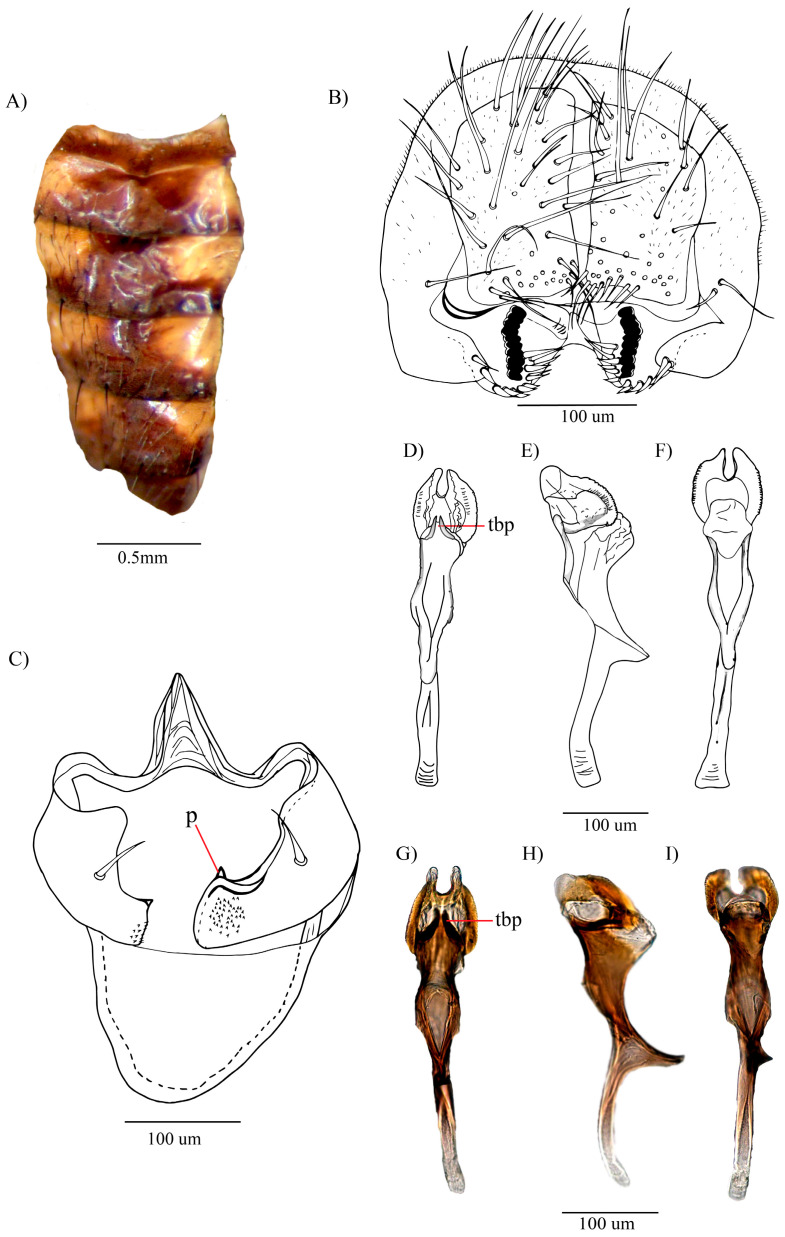
*Drosophila paraloewi* sp. nov., holotype ♂ (QCAZ-I 261171): (**A**) Abdomen in dorsal view. (**B**) External terminalia. (**C**) Hypandrium (paraphysis attached to hypandrium). (**D**–**F**) Aedeagus in dorsal, lateral, and ventral views, respectively (drawn from a paratype). (**G**–**I**) Pictures of the aedeagus in dorsal, lateral, and ventral views, respectively (tbp = triangular bifurcated projection; p = paraphysis).

**Abdomen.** Yellow. Tergite 1 with two brown shadows covering almost the entire segment. Tergites 2 and 3 each with a central hourglass-shaped, brown spot extending toward the posterior margins. Tergites 4 and 5 each with a triangular brown spot extending toward the posterior margins. Tergite 6 completely brown (Figure 3A).

**External terminalia**. Epandrium microtrichose. Cerci free. Ventral lobe with one seta present at the base of the surstylus. Surstylus with 10 prensisetae on both sides, the right side with 11 outer setae, and the left side with 10 outer setae (Figure 3B).

**Internal terminalia.** Hypandrium shield-shaped, with an elongated, striated dorsal arch. Gonopods microtrichose, each with a long seta. Paraphysis (p) attached to the gonopods, with a small seta (Figure 3C). Aedeagus chitinized and tubular, the distal part widened, with two chitinized, serrated laminae; the dorso-apical membrane pronounced, bifurcated, with refractive points resembling a serrated edge on the membrane; dorso-subapical with a chitinized, triangular, bifurcated projection; the ventral side, with a globose membrane with striations; ventral rod prominent. Apodeme chitinized (Figure 3D–I).

#### 3.2.3. Distribution

Known only from the type locality, Pasochoa, Pichincha, Ecuador, between 3260 and 3310 m above sea level.

#### 3.2.4. Biology

Unknown. The specimens were collected using banana-baited traps in the Eastern montane rainforest.

#### 3.2.5. Etymology

The specific name “*paraloewi*” refers to the morphological similarity of *Drosophila loewi.* The prefix “para” is used for less closely related organisms.

#### 3.2.6. Relationship

This species would be related to *D. loewi* Vilela and Bächli, 2000, and *D. hemiloewi* Suárez et al., 2024.

## 4. Discussion

**Figure 4:**
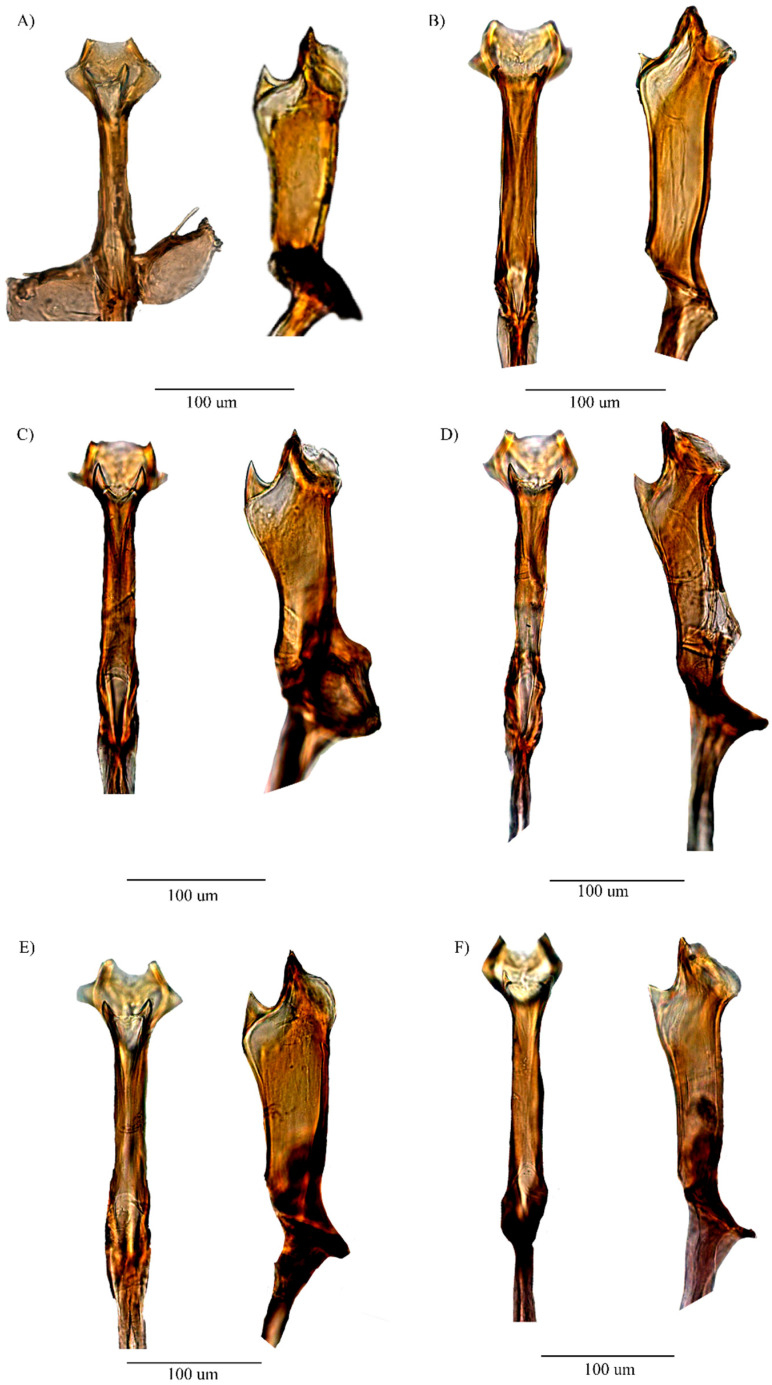
Variations of the aedeagus of *D. carlosvilelai*, dorsal and lateral views. (**A**) Holotype ♂ (QCAZ-I 261143). (**B**) Paratype ♂ (QCAZ-I 261144). (**C**) Paratype ♂ (QCAZ-I 261149). (**D**) Paratype ♂ (QCAZ-I 261151). (**E**) Paratype ♂ (QCAZ-I 261152). (**F**) Paratype ♂ (QCAZ-I 261153).

*D. carlosvilelai* was re-described based on a neotype newly designated, and the female morphology was described for the first time. Although minor variations in the arrangement of the aedeagal structure were observed between the neotype and other specimens examined (Figure 4), such variations were not sufficient to warrant the recognition of separate species. *D. carlosvilelai* and *Drosophila divisa* Duda, 1927, are closely related as both species lack inner setae on the ventral lobe of the epandrium and exhibit similar aedeagal structures. *D. divisa* shows a different number of prensisetae and outer setae compared to our neotype; however, these differences fall within the variations observed among the paratypes examined by Rafael and Vela [5]. In the female, the shape of the oviscapt, the number of outer ovisensilla, and the shape of the spermatheca correspond to the description of the *D. divisa* female collected from Peru, but differ from those of the paralectotypes from Bolivia [7]. These similarities may suggest possible conspecificity between *D. carlosvilelai* and *D. divisa*; however, further studies are needed to confirm this. Consequently, based on the aforementioned male and female morphological characteristics, *D. carlosvilelai* should be classified into subgroup III of the *tripunctata* group, along with *D. divisa*

**Figure 5:**
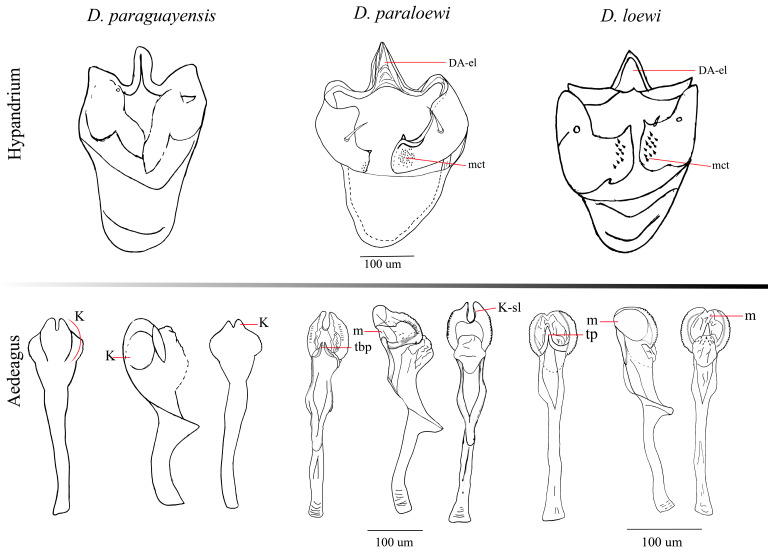
Comparison between the internal genital structures of *D. paraguayensis*, *D. paraloewi* sp. nov., and *D. loewi*. (DA-el = elongated dorsal arc, K-sl = slightly keratinized, m = membranous, mct = microtrichous, K = keratinized, tp = triangular projection, and tbp = triangular bifurcated projection). References: The schematic illustration of *Drosophila paraguayensis* was created based on the morphological description and figures from Vela and Rafael [5], and the images of *Drosophila loewi* hypandrium were based on the reference Vilela and Bächli [14], except for the aedeagus, which was drawn from a specimen collected in Ecuador.

The deep morphological analysis of *D. paraguayensis* registered in Ecuador in the year of 2001 [5] showed characters unrelated to its re-description [7]. The first difference encountered was the external morphological color of both species; *D. paraguayensis* is yellow, meanwhile *D. paraloewi* sp. nov. is brown. Additionally, in external terminalia, *D. paraguayensis* has the cerci not fused, unlike *D. paraloewi* sp. nov., which has them free. Both species have a similar number of prensisetae in a range of 8 to 10, no inner setae, and between 8 to 16 outer setae. The *D. paraguayensis* aedeagus bears two subapical, serrated lateral processes, and both the subapical ventral and dorsal surfaces are laterally membranous, covered with tiny spines, which is the basis of the misidentification [5,7]. However, *D. paraloewi* sp. nov. shares certain similarities with *D. paraguayensis* aedeagus, such as a distal part widened with two chitinized and serrated laminae and a bifurcated dorso-apical membrane that displays refractive points resembling a serrated edge on the membrane. Nevertheless, it has a unique character that resembles a structure from another well-known species, *D. loewi* (Figure 5), as well as in *D. hemiloewi,* a closely related species recently described [13]. Vilela and Bächli [7] placed *D. paraguayensis* in subgroup II; meanwhile, the same authors [14] placed *D. loewi* in subgroup IV of the *tripunctata* group. For this reason, we suggest that *D. paraloewi* sp. nov. belongs in subgroup IV, as *D. loewi* does. We cannot assert that both species, *D. paraguayensis* and *D. paraloewi* sp. nov., have a close relationship; for that, we need more studies on these species.

**Figure 6:**
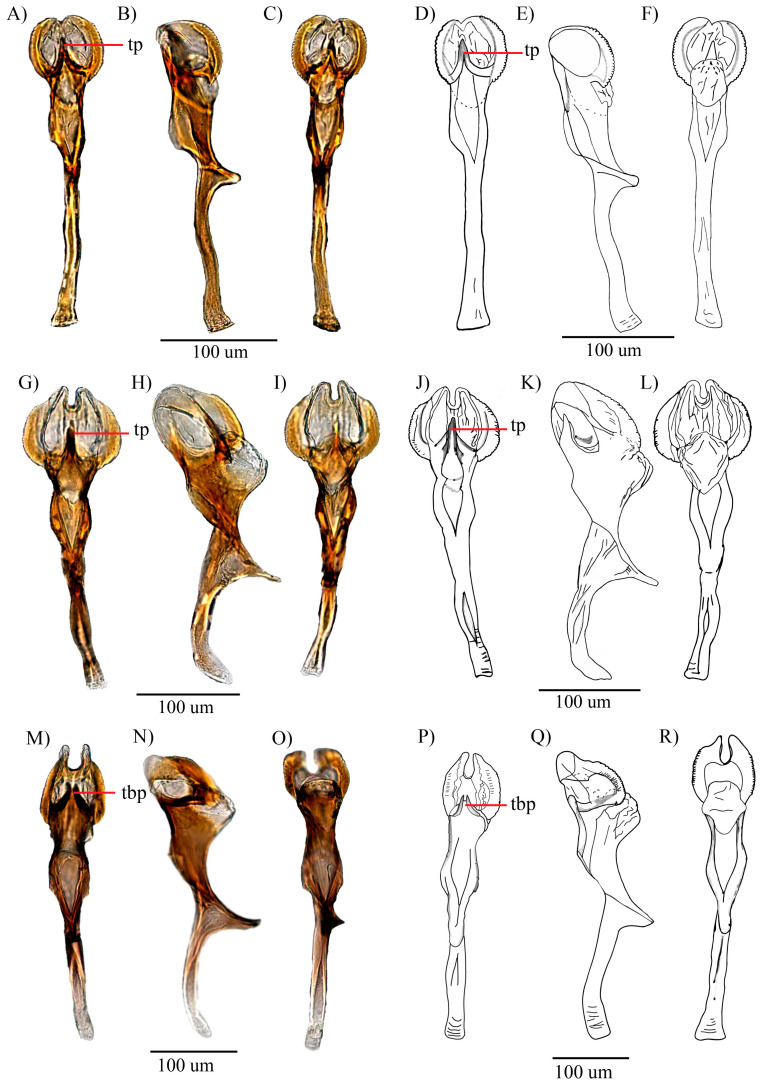
Aedeagus of D. *loewi* [14]-related species: *D. loewi* (**A**–**C**) pictures of the aedeagus in dorsal, lateral, and ventral views, and (**D**–**F**) drawings of the aedeagus in dorsal, lateral, and ventral views, respectively. *D. hemiloewi* (**G**–**I**) pictures of the aedeagus in dorsal, lateral, and ventral views, and (**J**–**L**) drawings of the aedeagus in dorsal, lateral, and ventral views, respectively. *D. paraloewi* sp. nov. (**M**–**O**) pictures of the aedeagus in dorsal, lateral, and ventral views, and (**P**–**R**) drawings of the aedeagus in dorsal, lateral, and ventral views, respectively. (tp = triangular projection; tbp = triangular bifurcated projection).

*D. loewi* has a triangular projection structure located between the two keratinized serrated laminae (tp), as indicated by Bächli et al. [15] (Figure 6), also found in *D. hemiloewi* and our type species *D. paraloewi* sp. nov., which would indicate that they are more closely related to *D. loewi* than to *D. paraguayensis. D. paraloewi* sp. nov., in contrast to *D. loewi*, possesses a single seta at the base of the right surstylus and two setae on the left surstylus. Additionally, the ventral lobes have the same rectangular shape but with two internal setae on each side. A more thorough analysis revealed that the hypandrium displays the same differences mentioned in the comparison between *D. hemiloewi* and *D. loewi* by Suárez [13]; *D. paraloewi* sp. nov. is similar to *D. loewi* in the shape of the aedeagus and the detailed structure of the head of the aedeagus. The head shape is more elongated in the dorsal view but flattened in the lateral view. Unlike *D. hemiloewi,* in which the membrane projects dorso-subapically, the membrane projects through the ear-shaped chitinized latero-distal membranes [12,15] (Table 1). Thus, *D. paraloewi* sp. nov. is most closely related to *D. loewi* and *D. hemiloewi*. Consequently, the previous record of *D. paraguayensis* in Ecuador has confirmed that the original identification was erroneous and that the specimen in question should be assigned to *D. paraloewi* sp. nov.

## Figures and Tables

**Table 1:** Comparison between internal genital structures of *D. loewi*, *D. hemiloewi*, and *D. paraloewi* sp. nov.

	Structure	*Drosophila loewi*	*Drosophila hemiloewi*	*Drosophila paraloewi* sp. nov.
External terminalia	Ventral lobe	Rectangular shaped with one inner seta	Rounded shaped with two inner setae	Rectangular shaped with two inner setae
	Surstylus	Three setae at the base on both sides	Single seta at the base, on the right side and three on the left side.	Single seta at the base, on the right side and two on the left side.
Internal terminalia	Hypandrium	Gonopod merged to paraphysis	Gonopod merged to paraphysis	Gonopod merged to paraphysis
		Lateral roughness on the submedial external surface of the gonopod	Lack lateral roughness on the submedial external surface of the gonopod	Lack lateral roughness on the submedial external surface of the gonopod
	Aedeagus apex	Head elongated in the dorsal view but flattened in the lateral view	Head ear-shaped chitinized latero-distal membranes projected in the dorso-subapical view	Head elongated in the dorsal view but flattened in the lateral view
		Triangular projection between the two keratinized, serrated laminae	Triangular projection between the two keratinized, serrated laminae	Triangular bifurcated projection between the two keratinized, serrated laminae

References: *D. hemiloewi* pictures G–I and drawings J–L were taken from the work of Suárez et al., [13].

## Data Availability

The original contributions presented in this study are included in the article. Further inquiries can be directed to the corresponding authors.

## Abbreviations

The following abbreviations are used in this manuscript:

MASL	Meters Above Sea Level
QCAZ-I	Quito Católica Zoología Sección Invertebrados
TPB	Triangular and bifurcated shape
P	Paraphysis
A-el	Elongated Dorsal Arc
K-sl	Slightly Keratinized
M	Membranous
MCT	Microtrichous
K	Keratinized
TP	Triangular projection
